# Fungal β-Glucans Shape Innate Immune Responses in Human Peripheral Blood Mononuclear Cells (PBMCs): An In Vitro Study on PRR Regulation, Cytokine Expression, and Oxidative Balance

**DOI:** 10.3390/ijms26136458

**Published:** 2025-07-04

**Authors:** Elżbieta Kozłowska, Justyna Agier, Sylwia Różalska, Magdalena Jurczak, Aleksandra Góralczyk-Bińkowska, Paulina Żelechowska

**Affiliations:** 1 Department of Microbiology, Genetics, and Experimental Immunology, Medical University of Lodz, 92-215 Lodz, Poland; justyna.agier@umed.lodz.pl (J.A.); magdalena.jurczak@umed.lodz.pl (M.J.); aleksandra.goralczyk-binkowska@umed.lodz.pl (A.G.-B.); paulina.zelechowska@umed.lodz.pl (P.Ż.); 2 Department of Industrial Microbiology and Biotechnology, University of Lodz, 90-237 Lodz, Poland; sylwia.rozalska@biol.uni.lodz.pl

**Keywords:** Dectin-1, Dectin-2, Toll-like receptor, reactive oxygen species, curdlan, zymosan, antioxidant enzymes

## Abstract

Fungi are ubiquitous organisms that are capable of transient or persistent colonization in humans. Their polymorphic nature and complex host–mycobiome interactions remain incompletely understood. Emerging evidence highlights the role of resident fungi in modulating immune responses and adapting to host changes, which can trigger a shift from commensalism to parasitism, particularly in immunocompromised individuals. This study evaluated the effects of two major β-glucans—zymosan and curdlan—on the expression of pattern recognition receptors (*Dectin1*, *Dectin2*, *TLR2*, *TLR4*) in human peripheral blood mononuclear cells (PBMCs). It also examined their impact on reactive oxygen species (ROS) production, cytokine/chemokine gene expression, and antioxidant enzyme expression. Both β-glucans significantly increased the mRNA levels of all tested receptors and enhanced ROS generation. Curdlan downregulated key antioxidant enzymes (*SOD1*, *CAT*, *GPX1*), while zymosan markedly upregulated *SOD1*. These findings demonstrate that the β-glucans zymosan and curdlan have a substantial influence on PBMC reactivity and oxidative stress responses. Further studies are needed to deepen our understanding of host–fungal interactions and their implications in health and disease.

## 1. Introduction

The mycobiome, a component of the microbiome community, comprises fungi shaped by their quantitative and qualitative composition, temporal or local variability, and the sum of interactions among them and between members of other subgroups, such as the bacteriome and virome. External factors, e.g., nutrients, alcohol, and medications, act as environmental variables that significantly impact this collectivity. The intestines have received the most attention regarding the human mycobiome; however, different areas, such as the skin, the respiratory tract, the vagina, and oral or nasal cavities, have also been inhabited [1,2]. In a healthy state, resident fungi and their host maintain a mutualistic relationship [1,2,3]. An expanding amount of data underscores the role of symbiotic fungi in shaping immunity by facilitating the development of peripheral lymphoid organs [4] and affecting immune cell responses [5,6]. Nevertheless, the nature of this connection is not as thoroughly understood, and fungi’s polymorphic nature is a crucial virulence factor that plays a vital role in the shift from relative tolerance to destruction [7].

The efficiency of the host defense system depends significantly on pattern recognition receptors (PRRs), which identify microbial signals known as pathogen-associated molecular patterns (PAMPs). Four major subfamilies of PRRs are currently acknowledged: cytoplasmic sensors, including RIG-I-like receptors (RLRs) and NOD-like receptors (NLRs), along with cell membrane-anchored Toll-like receptors (TLRs) and C-type lectin receptors (CLRs) [8]. Dectin-1, Dectin-2, Mincle, and Mannose Receptor (MR) from the CLR family, along with TLR2, TLR4, and TLR6 from the TLRs, are the most effective in sensing fungi. Alongside membrane receptors, soluble PRRs such as mannose-binding lectins (MBLs) derived from CLRs are essential for fungi detection [9]. The interaction of fungal components, especially carbohydrates like β-glucans, chitin, and mannose-based structures [10] with PRRs establishes the foundation for the immunological cascade [11]. The recognition of fungi by PRRs, particularly Dectin-1 and Dectin-2, enhances phagocytosis, increases the production of reactive oxygen species (ROS) [12], generates proinflammatory cytokines [13], and regulates Th1 and Th17 responses [14]. The structural differences between β-glucans used in this study are summarized in Table 1 [15,16].

In contrast to the bacterial microbiome, which remains relatively stable over time, the composition of the mycobiome varies significantly [1]. In certain circumstances, fungal colonizers can become Trojan horses, disrupting advantageous relationships with mammalian hosts. Fungi’s morphological plasticity contributes considerably to their success as both commensals and pathogens [7]. Several fungal genera are commonly found as commensals in the human microbiota, including *Candida*, *Malassezia*, *Saccharomyces*, and *Cladosporium* [17]. *Candida albicans* is the most well-characterized and prevalent fungal commensal, especially in the gastrointestinal and genitourinary tracts [18]. Under conditions of dysbiosis or immune suppression, some commensal fungi, such as *C. albicans*, can shift toward pathogenicity and cause localized or even systemic infections [19]. In contrast, other clinically relevant fungal pathogens, such as *Aspergillus fumigatus* or *Cryptococcus neoformans*, are not typically part of the human commensal mycobiota but can cause severe opportunistic infections, particularly in immunocompromised individuals [19]. Dectin-1 expression affects the makeup of the gut microbiota, and colonization of opportunistic pathogenic fungi that harm beneficial ones is one effect of receptor lack [20]. Also, during fungal dysbiosis, Dectin-1 regulates antifungal immunity to prevent the overgrowth of opportunistic fungi [21]. The limited number of research papers suggests that disturbances in the mycobiota composition may lead to the unfavorable cohabitation of bacteria and fungi, which could be associated with pathological conditions such as Crohn’s disease or cystic fibrosis [1,22]. The dysbiosis experiments demonstrate that the relationship between commensal fungi and the bacteriome is a delicate balance that can easily be disrupted. When the microbiome is unsettled, the commensal components of the mycobiome can become unregulated and pathogenic [22]. An imbalanced gut microbiome compromises the intestinal barrier and disrupts cellular immunity, especially neutrophils, leading to the translocation of intestinal-colonizing fungi across the epithelial barrier into the bloodstream [2,23].

Our previous report demonstrated that β-glucans and mannans, such as curdlan, zymosan, and mannan, can modulate the activity of peripheral blood mononuclear cells (PBMCs) by inducing the production of key cytokines and chemokines, including IFN-γ, CCL3, GM-CSF, IL-1β, and CXCL8. Additionally, our findings suggest that the Syk signaling pathway is involved in this response, as its inhibition significantly reduced cytokine and chemokine expression [24]. In light of these results, this study was designed to clarify the interaction of β-glucans (zymosan and curdlan) with PRRs in human PBMCs and examine the immune effects of this interaction.

## 2. Results

### 2.1. The Effect of Curdlan and Zymosan on TLR2, TLR4, Dectin1, and Dectin2 mRNA Expression on PBMCs

**Figure 1:**
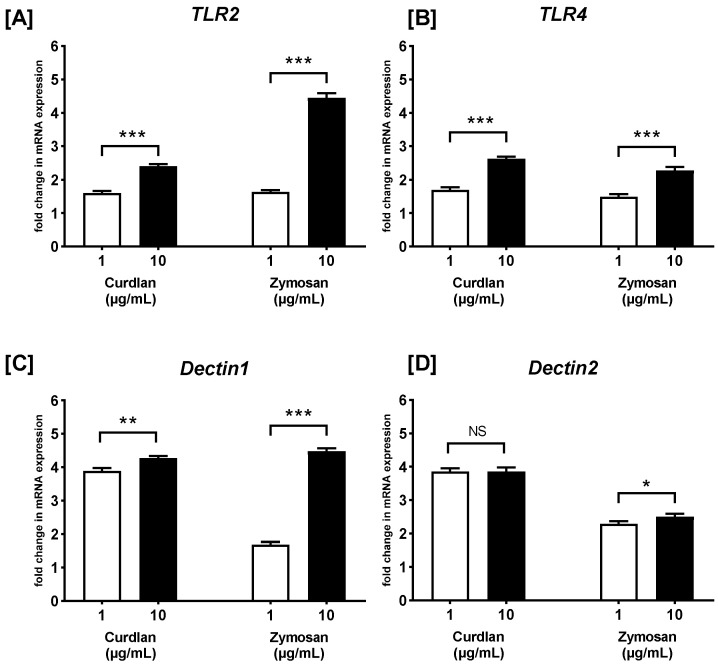
The effects of curdlan and zymosan on (**A**) *TLR2*, (**B**) *TLR4*, (**C**) *Dectin1*, and (**D**) *Dectin2* mRNA expression in PBMCs, evaluated by RT-qPCR. PBMCs were incubated with curdlan and zymosan at 1 or 10 μg/mL concentrations or with medium alone for 72 h. Total mRNA was extracted, and cDNA was synthesized from mRNA. RT-qPCR was then performed to evaluate the expression levels of the four receptors. The expression levels were normalized to the transcript level of the housekeeping gene *ACTB*, and the relative mRNA expression was calculated using the 2^−ΔΔCq^ method. The results are presented as the mean ± SD of three independent experiments conducted in duplicate. * *p* < 0.05, ** *p* < 0.01, *** *p* < 0.001; NS—not significant.

Firstly, we examined the effect of both β-glucans (at 1 μg/mL and 10 μg/mL) on the mRNA expression of *TLR2*, *TLR4*, *Dectin1*, and *Dectin2* on PBMCs (Figure 1). As calculated after RT-qPCR, curdlan induced an increase in the mRNA expression level of *TLR2* (1.6- and 2.4-fold; both *p* > 0.05) (Figure 1A), *TLR4* (1.7- and 2.6-fold; *p* < 0.01 and *p* > 0.05, respectively) (Figure 1B), *Dectin1* (3.9- and 4.3-fold; both *p* > 0.05) (Figure 1C), and *Dectin2* (3.9- and 3.9-fold; both *p* > 0.05) (Figure 1D) in PBMCs stimulated with 1 and 10 µg/mL, respectively, when compared to non-treated control cells. Similarly, zymosan also increased the mRNA expression levels of *TLR2* (1.6- and 4.5-fold; *p* > 0.05 and *p* < 0.05, respectively) (Figure 1A), *TLR4* (1.5- and 2.3-fold; both *p* < 0.05) (Figure 1B), *Dectin1* (1.7- and 4.5-fold; *p* < 0.05, *p* < 0.0001, respectively) (Figure 1C), and *Dectin2* (2.3- and 2.5-fold; *p* < 0.05, *p* < 0.01, respectively) (Figure 1D) in PBMCs stimulated with 1 and 10 µg/mL, respectively, when compared to unstimulated control cells. In the case of both stimulants, the concentration of 10 µg/mL induced a statistically significantly greater increase in the mRNA expression of the investigated receptors than the concentration of 1 µg/mL, except for the mRNA expression of *Dectin2*. Both concentrations of curdlan—1 and 10 µg/mL—induced a similar increase in *Dectin2* mRNA expression.

### 2.2. The Effects of R406, SB203580, Laminarin, and TAK-242 on Curdlan- and Zymosan-Induced TLR2, TLR4, Dectin1, and Dectin2 mRNA Expression on PBMCs

**Figure 2:**
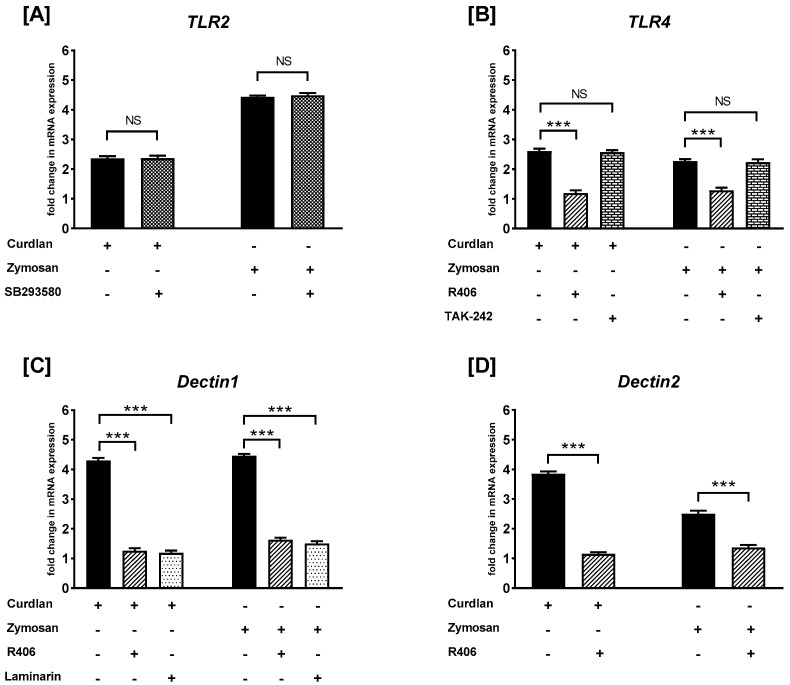
The effects of R406, SB203580, laminarin, and TAK-242 on curdlan- and zymosan-induced (**A**) *TLR2*, (**B**) *TLR4*, (**C**) *Dectin1*, and (**D**) *Dectin2* mRNA expression in PBMCs, evaluated by RT-qPCR. PBMCs were preincubated with R406 at 2 μM, SB203580 at 10 μM, laminarin at 100 μg/mL, TAK-242 at 5 ng/mL, or medium alone for 30 min before stimulation with curdlan or zymosan at 10 μg/mL. Total mRNA was extracted and used for cDNA synthesis. RT-qPCR was conducted to assess the expression of the receptors at the mRNA level. The expression levels were normalized to the transcript level of the housekeeping gene *ACTB*, and relative mRNA expression was calculated using the 2^−ΔΔCq^ method. The results are presented as the mean ± SD of three separate experiments performed in duplicate. *** *p* < 0.001; NS—not significant.

To examine whether the studied receptors are involved in the β-glucan-mediated response in PBMCs, SB203580, as well as the p38 MAPK inhibitor, R406, Syk kinase inhibitor, TAK-242, TLR4 antagonist, and laminarin, Dectin-1 antagonist were used (Figure 2). We observed that PBMC pretreatment with SB203580 did not affect (*p* > 0.05) the curdlan- and zymosan-stimulated mRNA levels of *TLR2* (Figure 2A). In turn, pretreatment of PBMCs with R406 caused statistically significant suppression (*p* < 0.001) of the curdlan- and zymosan-stimulated mRNA expression levels of *TLR4*; however, TAK-242 did not affect the level of mRNA expression induced by curdlan or zymosan (Figure 2B). We also demonstrated that PBMC pretreated with R406 or laminarin resulted in statistically significant suppression of curdlan- and zymosan-mediated *Dectin1* (*p* < 0.01) (Figure 2C) and *Dectin2* (*p* < 0.001) (Figure 2D) mRNA expression when compared to non-primed PBMCs.

### 2.3. The Effects of Curdlan and Zymosan on PBMC Cytokine/Chemokine mRNA Expression

**Figure 3:**
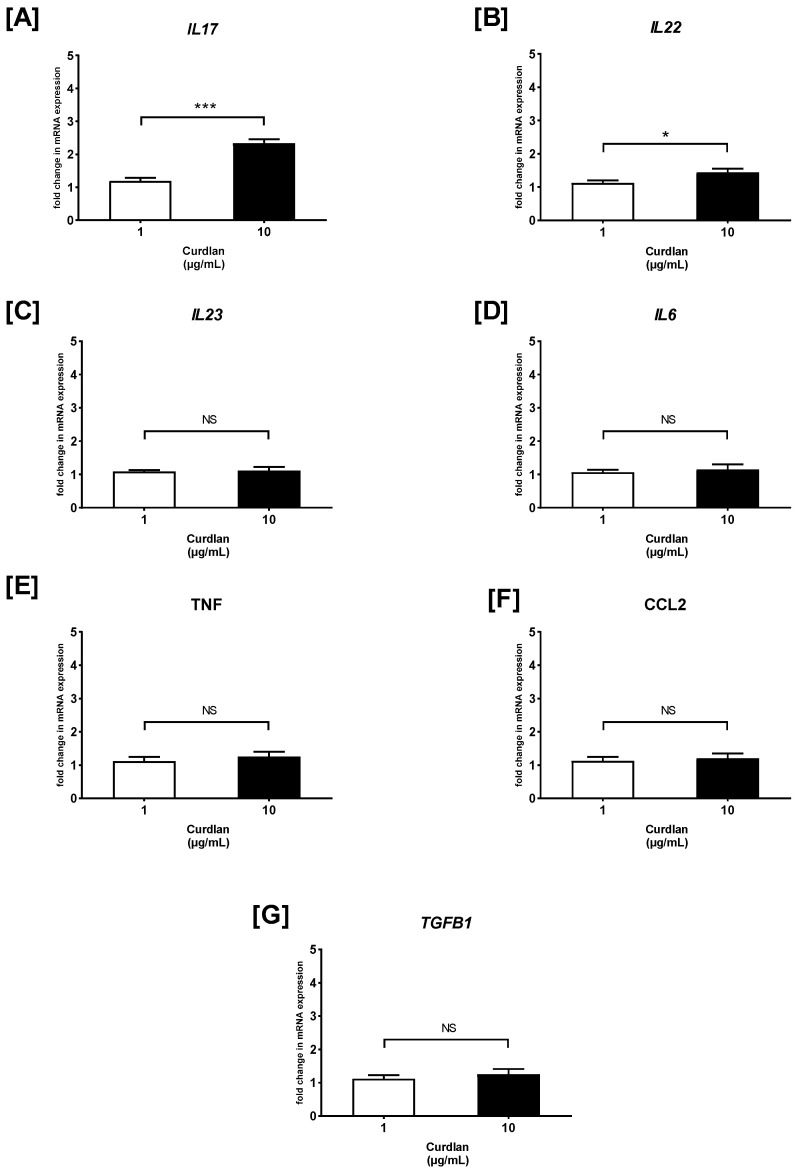
The effects of curdlan on (**A**) *IL17*, (**B**) *IL22*, (**C**) *IL23*, (**D**) *IL6*, (**E**) *TNF*, (**F**) *CCL2*, and (**G**) *TGFB1* mRNA expression in PBMCs evaluated by RT-qPCR. In the first step, PBMCs were cultured for 72 h with curdlan at 1 and 10 μg/mL or medium alone. Afterwards, total mRNA was extracted and used for cDNA synthesis, followed by a RT-qPCR assay. The expression levels of genes encoding selected cytokines/chemokines were normalized to the transcript level of the housekeeping gene *ACTB*, and relative mRNA expression was calculated using the 2^−ΔΔCq^ method. The results are shown as the mean ± SD of three separate experiments performed in duplicate. * *p* < 0.05, *** *p* < 0.001; NS—not significant.

**Figure 4:**
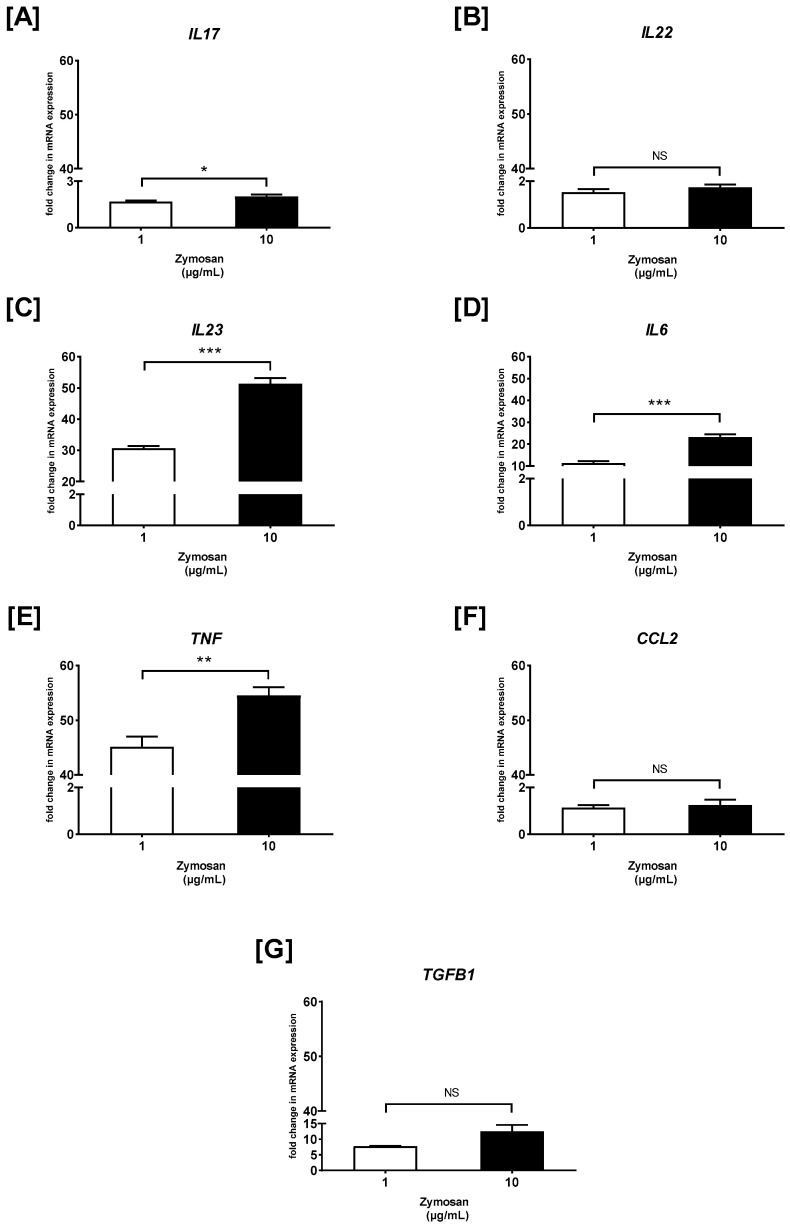
The effects of zymosan on (**A**) *IL17*, (**B**) *IL22*, (**C**) *IL23*, (**D**) *IL6*, (**E**) *TNF*, (**F**) *CCL2*, and (**G**) *TGFB1* mRNA expression in PBMCs evaluated by RT-qPCR. In the first step, PBMCs were cultured for 72 h with zymosan at 1 and 10 μg/mL or medium alone. Afterwards, total mRNA was extracted and used for cDNA synthesis, followed by a RT-qPCR assay. The expression levels of genes encoding selected cytokines/chemokines were normalized to the transcript level of the housekeeping gene *ACTB*, and relative mRNA expression was calculated using the 2^−ΔΔCq^ method. The results are shown as the mean ± SD of three separate experiments performed in duplicate. * *p* < 0.05, ** *p* < 0.01, *** *p* < 0.001; NS—not significant.

Next, RT-qPCR was carried out, and the fold change of cytokine/chemokine mRNA expression in curdlan- and zymosan-stimulated PBMCs compared to the non-stimulated cells was assessed (Figure 3 and Figure 4). We observed that the *IL17* mRNA levels were 1.2- and 2.3-fold higher in the PBMCs stimulated with 1 and 10 µg/mL of curdlan, respectively, when compared to the non-treated control cells (both *p* > 0.05) (Figure 3A). Moreover, the mRNA levels for *IL17* were slightly upregulated when the cells were exposed to 1 and 10 µg/mL of zymosan, as follows: 1.6- and 2-fold (both *p* > 0.05) (Figure 4A). *IL22* gene expression was narrowly increased from 1.1 to 1.4 times in the PBMCs challenged with 1 and 10 µg/mL of curdlan (both *p* > 0.05) (Figure 3B), while the increase only from 1.5 to 1.7 times was induced after stimulation with zymosan at 1 and 10 µg/mL (both *p* > 0.05) (Figure 4B). As can be seen in Figure 3C, the increase in the level of *IL23* mRNA was insubstantial when the PBMCs were treated with 1 and 10 µg/mL of curdlan (1.1- and 1.2-fold; both *p* > 0.05). In contrast, exposure of the PBMCs to zymosan at concentrations of 1 and 10 µg/mL resulted in very significant *IL23* mRNA expression upregulation (31- and 51-fold; both *p* > 0.05) in comparison to the non-stimulated cells (Figure 4C). We also found that the levels of *IL6* transcription in the PBMCs stimulated with 1 and 10 µg/mL of curdlan were comparable to unstimulated cells (1.1- and 1.2-fold; both *p* > 0.05) (Figure 3D), while the relevant increase from 11 to 23 times was induced after stimulating the PBMCs with zymosan at 1 (*p* > 0.05) and 10 µg/mL (*p* < 0.05) (Figure 4D). As in the case of *IL6*, we documented that the mRNA expression level of *TNF* in PBMCs treated with 1 and 10 µg/mL of curdlan were comparable to unstimulated cells (1.1- and 1.2-fold; both *p* > 0.05) (Figure 3E), while exposure of these cells to zymosan at a concentration of 1 and 10 µg/mL resulted in the most considerable among all tested cytokines/chemokines’ *TNF* mRNA expression upregulation (45- and 55-fold; both *p* < 0.05) (Figure 4E). For both the PBMCs treated with curdlan and zymosan at concentrations of 1 and 10 µg/mL, we documented no change in *CCL2* mRNA expression (1.1- and 1.2-fold; both *p* < 0.05) compared to the cells untreated with these stimulants (Figure 3F and Figure 4F). Simultaneously, in the case of the PBMCs being challenged with 1 and 10 µg/mL of curdlan, we observed a slight enhancement of *TGFB1* mRNA transcript levels (1.1- and 1.25-fold; both *p* < 0.05) (Figure 3G); however, the PBMCs treated with zymosan at concentrations of 1 and 10 µg/mL resulted in an 8- and 13-fold increase (both *p* < 0.05) of *TGFB1* mRNA expression, respectively (Figure 4G). In the case of curdlan, a concentration of 10 µg/mL induced a statistically significantly greater increase in the mRNA expression of the investigated mediators than a concentration of 1 µg/mL for *IL17* (*p* < 0.001) and *IL22* (*p* < 0.01). Additionally, we observed this relationship for *IL6*, *IL23* (*p* < 0.001), *TNF* (*p* < 0.01), and *IL17* (*p* < 0.05) after stimulation with zymosan.

### 2.4. The Effects of R406 and Laminarin on Curdlan- and Zymosan-Induced PBMC Responses

**Figure 5:**
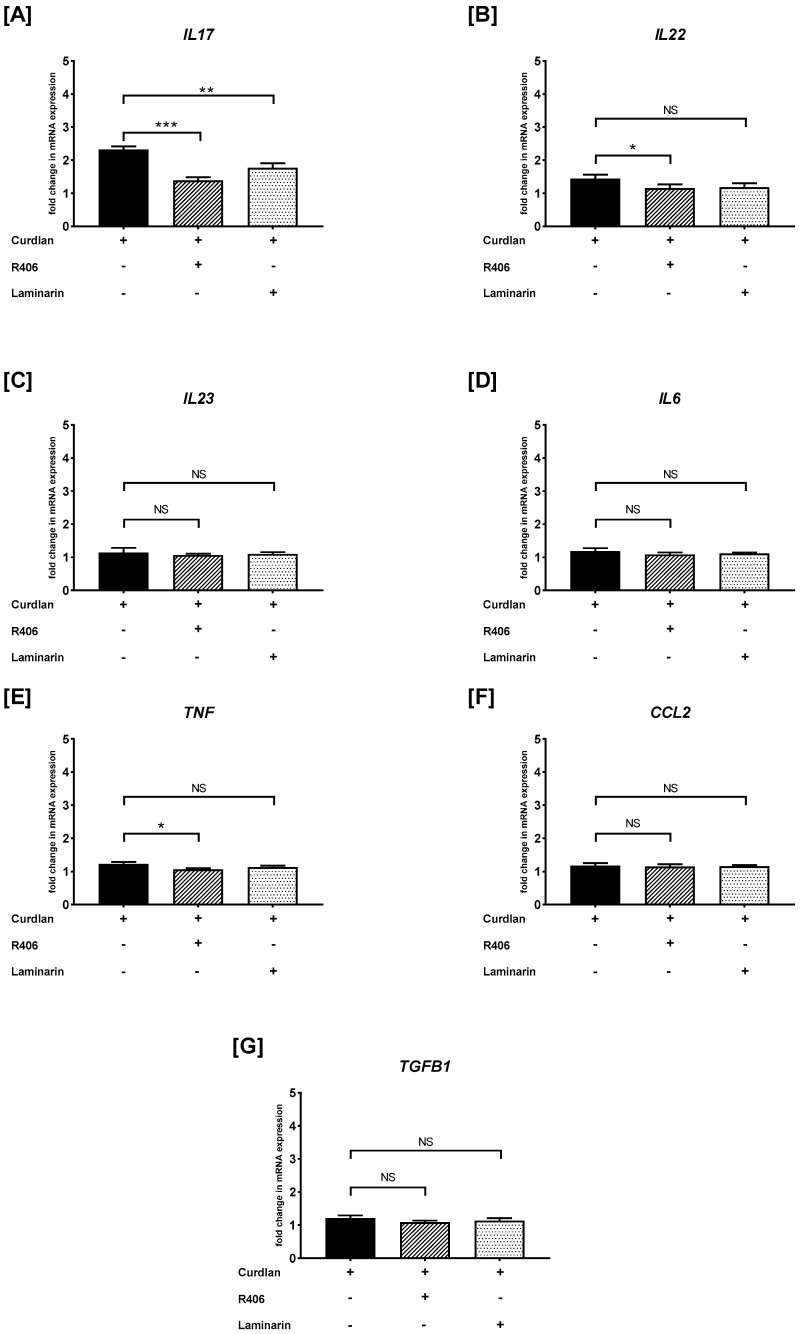
The effects of R406 and/or laminarin on curdlan-induced (**A**) *IL17*, (**B**) *IL22*, (**C**) *IL23*, (**D**) *IL6*, (**E**) *TNF*, (**F**) *CCL2*, and (**G**) *TGFB1* mRNA expressions in PBMCs. PBMCs were pretreated for 30 min with R406 at 2 μM or laminarin at 100 μg/mL, or medium alone, before stimulation with curdlan at a concentration of 10 μg/mL. Total mRNA was extracted and used for cDNA synthesis, followed by RT-qPCR to evaluate cytokine and chemokine mRNA expression. The expression levels were normalized to the transcript level of the housekeeping gene *ACTB*, and relative mRNA expression was calculated using the 2^−ΔΔCq^ method. The results are shown as the mean ± SD of three separate experiments performed in duplicate. * *p* < 0.05, ** *p* < 0.01, *** *p* < 0.001; NS—not significant.

As shown in Figure 5A, the PBMCs preincubated with R406 or laminarin caused a substantial suppression of curdlan-induced *IL17* mRNA expression. We also documented that the PBMCs preincubated with R406, but not with laminarin, reduced the curdlan-mediated mRNA levels of *IL22* and *TNF* (Figure 5B,E). Additionally, we established that pre-stimulation of PBMCs with R406 or laminarin did not lead to inhibition in the curdlan-mediated mRNA expression of *IL23* (Figure 5C), *IL6* (Figure 5D), *CCL2* (Figure 5F), and *TGFB1* (Figure 5G).

**Figure 6:**
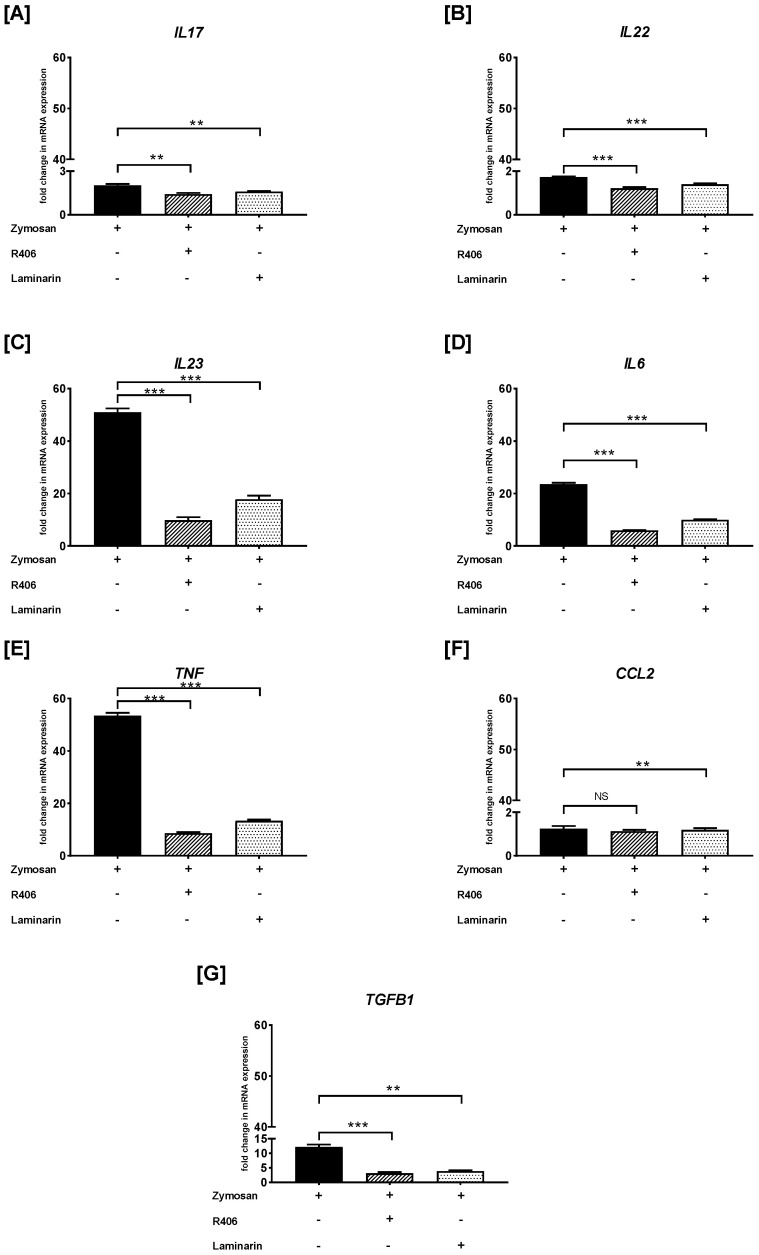
The effects of R406 and/or laminarin on zymosan-induced (**A**) *IL17*, (**B**) *IL22*, (**C**) *IL23*, (**D**) *IL6*, (**E**) *TNF*, (**F**) *CCL2*, and (**G**) *TGFB1* mRNA expression in PBMCs. PBMCs were pretreated for 30 min with R406 at 2 μM or laminarin at 100 μg/mL, or medium alone for 30 min before stimulation with zymosan at a concentration of 10 μg/mL. Total mRNA was extracted and used for cDNA synthesis, followed by RT-qPCR to evaluate cytokine and chemokine mRNA expression. The expression levels were normalized to the transcript level of the housekeeping gene *ACTB*, and relative mRNA expression was calculated using the 2^−ΔΔCq^ method. The results are shown as the mean ± SD of three separate experiments performed in duplicate. ** *p* < 0.01, *** *p* < 0.001; NS—not significant.

Similarly, we examined the effects of R406 and laminarin on the zymosan-induced PBMC response (Figure 6). We found that preincubation of PBMCs with either R406 or laminarin significantly reduced zymosan-induced *IL17* mRNA synthesis (*p* < 0.01) (Figure 6A). The most potent inhibition of zymosan-mediated mRNA expression after pretreatment with R406 and laminarin was observed for *IL22* (*p* < 0.001) (Figure 6B), *IL23* (*p* < 0.001) (Figure 6C), *IL6* (*p* < 0.001) (Figure 6D), and *TNF* (*p* < 0.001) (Figure 6E). Conversely, PBMCs preincubated with laminarin, but not with R406, resulted in a statistically significant reduction (*p* < 0.01) in zymosan-stimulated mRNA expression of *CCL2* (Figure 6F). Furthermore, the level of zymosan-induced *TGFB1* mRNA transcript was decreased in cells pretreated with R406 (*p* < 0.001) and laminarin (*p* < 0.01) (Figure 6G).

### 2.5. The Impact of Curdlan and Zymosan on ROS Production by PBMCs

**Figure 7:**
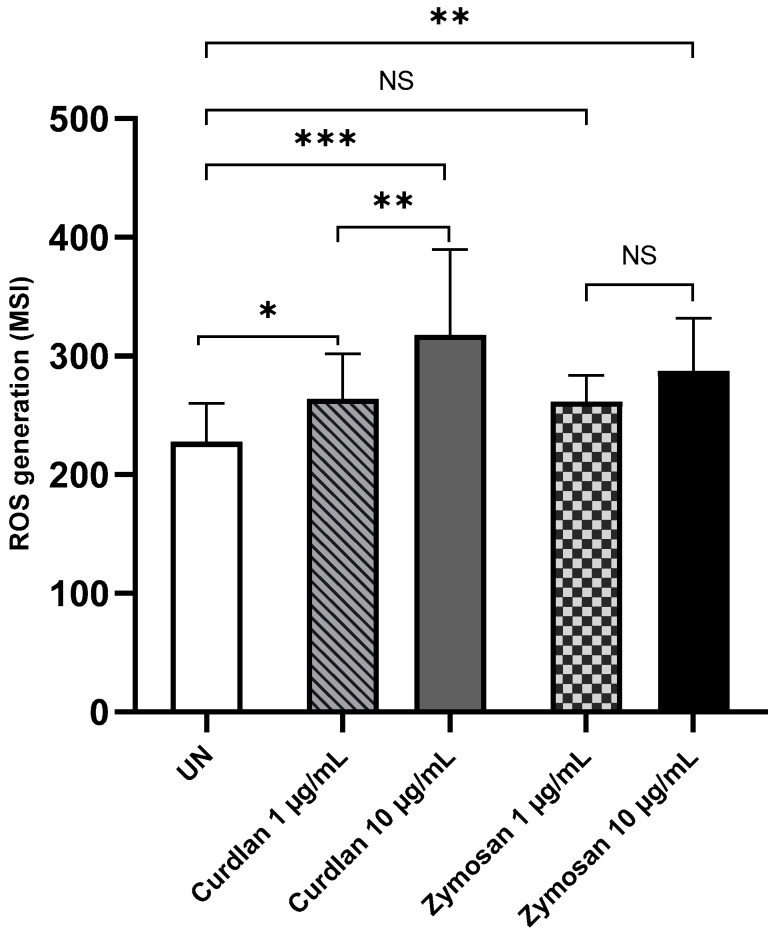
Curdlan- and zymosan-induced ROS production by PBMCs. PBMCs were incubated with curdlan and zymosan at 1 and 10 μg/mL or medium alone for 72 h. CellROX^™^ Green Reagent was used at a concentration of 5 μM for 30 min. The results are shown as the mean ± SD of four independent experiments, and each experiment was carried out in duplicate samples. UN—unstimulated cells; * *p* < 0.05, ** *p* < 0.01, *** *p* < 0.0001; NS—not significant.

Next, we examined the production of ROS by PBMCs in response to β-glucans. As shown in Figure 7, the stimulation of PBMCs with curdlan and zymosan significantly increased ROS generation compared to the non-stimulated cells. The statistical analysis revealed that ROS production in PBMCs stimulated with curdlan at 1 and 10 µg/mL doses was significantly higher than that in unstimulated PBMCs (*p* < 0.05 and *p* < 0.0001, respectively). Moreover, statistically significant increases in ROS production were observed in PBMCs stimulated with zymosan at concentrations of 10 µg/mL compared to the unstimulated control cells (*p* < 0.001).

### 2.6. The Effects of Curdlan and Zymosan on SOD1, CAT, and GPX1 mRNA Expression on PBMCs

**Figure 8:**
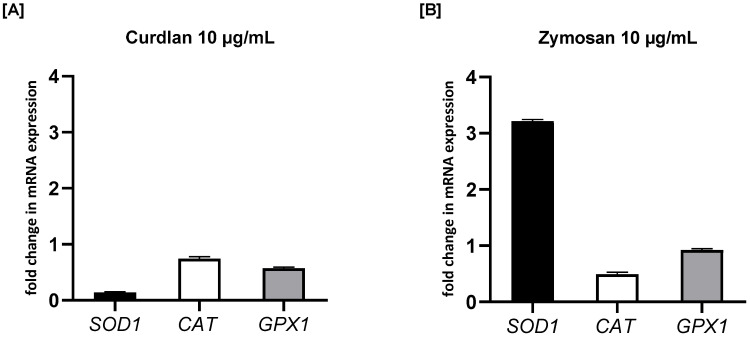
The effects of (**A**) curdlan and (**B**) zymosan on *SOD1*, *CAT*, and *GPX1* mRNA expression in PBMCs evaluated by RT-qPCR. PBMCs were incubated with curdlan or zymosan at 10 μg/mL or with medium alone for 72 h. Total mRNA was extracted and used for cDNA synthesis, followed by RT-qPCR to assess the expression of the three enzymes at the mRNA level. The expression levels were normalized to the transcript level of the housekeeping gene *ACTB*, and relative mRNA expression was calculated using the 2^−ΔΔCq^ method. The results are presented as the mean ± SD of three independent experiments conducted in duplicate.

To investigate the effects of β-glucans on oxidative stress-related enzymes, we measured the mRNA expression levels of *SOD1*, *CAT*, and *GPX1* in PBMCs treated with curdlan and zymosan at 10 μg/mL. As illustrated in Figure 8, treatment with curdlan resulted in a reduction in the expression of all three analyzed antioxidant enzymes compared to the untreated control. Specifically, the expression of *SOD1* was reduced to 0.57-fold (*p* > 0.05), *CAT* to 0.74-fold (*p* > 0.05), and *GPX1* to 0.14-fold (*p* > 0.05) in response to curdlan stimulation. In contrast, zymosan stimulation resulted in varying effects on these genes. While the expression of *GPX1* was only slightly decreased (0.92-fold; *p* > 0.05) and *CAT* was significantly downregulated (0.49-fold; *p* > 0.05), a remarkable increase was seen in *SOD1* expression, which was upregulated to 3.22-fold (*p* < 0.01) compared to the untreated control. This indicates that zymosan significantly activates *SOD1* expression while simultaneously decreasing *CAT* and moderately affecting *GPX1.*

## 3. Discussion

Our understanding of the interactions between fungi and immune cells remains comparatively limited. Molecules derived from fungi that modulate immune responses, specifically β-glucans, mannans, and chitin, demonstrate multidirectional effects through PRRs, influencing phagocytosis, ROS production, and the secretion of various cytokines from immune cells in both humans and rodents [11], as documented in our previous studies [24,25,26,27,28]. These immunologically active molecules are found in the cell walls of various pathogenic fungi, including *Candida albicans* (β-glucans, mannans), *Aspergillus fumigatus* (β-glucans, chitin), and *Cryptococcus neoformans* (β-glucans, chitin) [29,30]. The most well-known interaction occurs through transmembrane Dectin-1, which specifically recognizes β-(1,3)(1,6)-D-glucans [31]. The identification of β-glucans through Dectin-1 induces epigenetic modifications within immune cells, resulting in a more effective response during infections, a phenomenon commonly referred to as trained immunity [13]. There is a considerable deficiency in knowledge regarding Dectin-2’s role in recognizing fungal molecular patterns. The specific ligand associated with Dectin-2 remains unidentified; however, it is likely to interact with the α-mannans present in *Candida albicans* [32]. Equally significant are the TLRs that identify zymosan through TLR2, mannan via TLR4, and chitin, which both TLR2 and TLR4 recognize [11].

In this study, we examined the influence of β-glucans, specifically curdlan and zymosan, on freshly isolated human PBMCs. Initially, we examined their impact on the expression of *Dectin1*, *Dectin2*, *TLR2*, and *TLR4* in PBMCs. A significant finding of this study was that curdlan and zymosan, in concentrations of 1 μg/mL or 10 μg/mL, enhanced the mRNA expression levels of all examined receptors in the respective cell type. These data follow, to some extent, what Li et al. [33] discovered: insoluble β-glucan from the *Candida albicans* cell wall (100 µg/mL) and zymosan (50 µg/mL) increased the mRNA expression of *Dectin1* in human THP-1 monocytes. Zymosan also enhanced the *TLR2* mRNA expression in these cells; however, no change in the *TLR2* mRNA levels was observed after treatment with insoluble β-glucan. Our results align with Glaser et al.’s [34] notification that increased *TLR2* expression in monocytes stimulated with zymosan at a 1 µg/mL concentration. Bonfim et al. [35] confirmed this finding after stimulating monocytes with zymosan at 10 μg/mL, with a focus on *TLR2* and *Dectin1*. Apetrei et al. [36] documented that curdlan can enhance the expression of *Dectin1* on monocytes at a concentration of 20 μg/mL.

Activating specific cell signaling pathways via antigen–receptor interactions represents a fundamental mode of cell communication. A wide range of PRRs, such as some members of the TLR and CLR families, play an essential role in antifungal immunity, and representatives from both receptor subtypes may interact to recognize specific fungal cell wall components. It should be underlined that Dectin-1 and TLR2 are very closely situated in the membrane surface of monocytes or macrophages and could synergistically affect human PBMCs [37]. Some authors suggest that the signaling pathways of Dectin-1 cooperate with TLR2 in recognizing β-glucans [38,39,40]. We found a noticeable boost in β-glucan-mediated *TLR2* mRNA expression levels, and we also saw similar encouraging results with *Dectin1* expression. These results and those previously mentioned substantiate that relatively low concentrations of fungal antigens enhance cellular sensitivity and may trigger a signal for immune hyperactivation. The cooperation of the signaling pathways related to these receptors may lead to faster and more effective recognition of fungal antigens and a more robust immune response, promoting the development of inflammation.

Laminarin acts as a Dectin-1 antagonist in various cell types. Our study demonstrates that PBMCs preincubated with laminarin and/or a Syk kinase inhibitor, a key molecule in CLR-mediated cell responses, downregulated β-glucan-induced *Dectin1* and *Dectin2* mRNA expression. These findings may confirm the participation of the studied β-glucans in enhancing the expression of *Dectin1* and *Dectin2* on the surface of immunocompetent cells. Previously, laminarin has been shown to reduce the zymosan-mediated surface expression of *Dectin1* on cultured bone marrow-derived murine mast cells [41].

The responsiveness of various cell populations to β-glucans may depend on the specific local environment in which they are activated [42]. Experimental data indicate that fungi such as *Candida albicans* can alter immune responses by modulating the reactivity of immunocompetent cells [43]. It is unequivocal that cytokines and chemokines play a pivotal role in orchestrating numerous antifungal defense mechanisms within the host [44]. They facilitate the mobilization of immune cells to the site of infection, enhance phagocytosis, and regulate the mechanisms associated with acquired immunity. For instance, TNF and IL-17 are crucial for maintaining the activity of neutrophils and macrophages [43,45].

Furthermore, it has been shown that IL-17 plays a vital role in the antifungal defense of mucous membranes, while IL-23 enhances its production by T lymphocytes [46,47]. IL-22, synthesized by Th17 cells, contributes to host defense and protects against candidiasis [34]. TGF-β is necessary for the differentiation of Th17 cells [43]. There is also evidence that CCL2 and IL-6 affect defensive strategies against fungi [48,49]. In a compelling study conducted by Quintin et al. [50], it was demonstrated that *C. albicans* and fungal cell wall β-glucans triggered the functional reprogramming of monocytes, leading to an increased production of cytokines, both in vivo and in vitro. Thus, it has been demonstrated that monocytes can be trained to exhibit an enhanced and prolonged response to microbial components following prior exposure to *C. albicans* or β-glucans. Additionally, specific cytokines such as TNF, IL-6, IL-1, and CXCL8 were secreted by human dendritic cells (DCs), monocytes/macrophages, and neutrophils in response to β-glucans [39,51,52,53,54,55].

Considering that cytokines and chemokines serve as essential humoral agents that affect cellular activity, it is crucial to investigate whether components associated with fungi modify the gene expression levels of multiple cytokines and chemokines, particularly those engaged in antifungal defense, in PBMCs. In the present study, we documented that curdlan induces a modest increase in the mRNA expression of *IL17*, *IL22*, *IL23*, *IL6*, *TNF*, *CCL2*, and *TGFB1*, with the most notable increase observed for *IL17*. At the same time, curdlan also caused a decrease in the expression of antioxidant enzymes, including *SOD1*, *CAT*, and *GPX1*. Based on these findings, curdlan may exert immunomodulatory effects in vitro, suggesting its potential relevance in conditions characterized by excessive inflammation. However, further studies are needed to explore its role in disease-specific contexts.

Dillon et al. [56] demonstrated that zymosan, acting through TLR2 and Dectin-1, modulates DCs’ IL-10 secretion and alters macrophage TGF-β production. According to research, the cooperative activity of these receptors also increases the production of proinflammatory cytokines, including TNF, IL-6, and IL-1β, by immunocompetent cells or enhances the phagocytic activity of macrophages [57,58,59]. Here, we confirmed this observation in the case of a PBMC challenge with zymosan, as we showed increased mRNA expression levels of both *TNF* and *IL6*. Furthermore, zymosan triggers a significant rise in mRNA levels for *IL23*, along with an exceptional *TGFB1* level.

We also demonstrated that zymosan induces expression changes more significantly than curdlan; we noted the highest increase in *IL23* and *TNF* expression. Other reports suggest that alveolar macrophages can produce TNF in response to zymosan depletion [60]. De Graaff et al. [15] noted that when human M(IL-4) macrophages were exposed to zymosan (500 μg/mL), *CCL2* mRNA expression remained unchanged; however, the secretion of IL-6 and TNF increased. They also demonstrated that following exposure to curdlan, the secretion of IL-6 and TNF from human M(IL-4) macrophages was elevated.

Our study also demonstrated that zymosan elicited an increase in ROS production, coinciding with an upregulation of *SOD1* expression. This observation suggests the activation of compensatory mechanisms within the antioxidant system. This study provides further evidence that β-glucans induce PBMC responses in a manner dependent on Dectin-1. Pre-stimulation of PBMCs with laminarin, which acts as a Dectin-1 antagonist, inhibited the expression of the studied cytokine transcripts, particularly in response to zymosan.

In conclusion, our investigation has yielded substantial evidence that model β-glucans—zymosan and curdlan—significantly influence the reactivity of human PBMCs. Our research demonstrates that zymosan elicits a more pronounced immune response compared to curdlan, as evidenced by an increase in cytokine mRNA transcripts for *IL23*, *IL6*, *TNF*, and *TGFB1*, accompanied by a marked elevation in ROS production. These cytokines are known to participate in inflammatory and antifungal immune responses, and their expression in PBMCs highlights the immunomodulatory potential of β-glucans. Additional research is imperative to elucidate the role of these molecules in regulating innate immune function. A more comprehensive understanding of fungal–host interactions may facilitate the advancement of preventive strategies or more efficacious therapies, particularly for individuals in whom dysregulation of the mycobiome contributes to disease manifestation.

## 4. Materials and Methods

### 4.1. Preparation of Peripheral Blood Mononuclear Cells (PBMCs)

PBMCs were isolated by centrifugation through Histopaque-1077 (Sigma-Aldrich, Saint Louis, MO, USA) from buffy coats obtained from healthy, anonymous donors, received as waste material from the Regional Center for Blood Donation and Blood Treatment in Lodz, Poland. Four milliliters of buffy coat were gently mixed with 5 mL of phosphate-buffered saline (PBS, 1×; Santa Cruz Biotechnology, Dallas, TX, USA) containing 2 mM ethylenediaminetetraacetic acid (EDTA, pH 8.0; Sigma-Aldrich) and were carefully layered onto 5 mL of Histopaque-1077. The PBS/EDTA buffer was prepared by combining 50 mL of 10× PBS, 2 mL of 0.5 M EDTA, and 448 mL of distilled water, resulting in final concentrations of 1× PBS and 2 mM EDTA. The cells were centrifuged at room temperature at 400× *g* for 30 min. The second opaque layer was aspirated and transferred directly to a clean tube, then washed three times with the previously prepared PBS/EDTA buffer (1× PBS containing 2 mM EDTA). A Bürker chamber (Marienfeld Superior™, Lauda-Königshofen, Germany) was utilized to count the number of PBMCs obtained. This study was conducted in accordance with the ethical standards outlined in the Declaration of Helsinki, as established by the World Medical Association (1964) and its subsequent amendments, and received approval from the Committee for Bioethics in Medicine at the Medical University of Lodz, Poland (RNN/39/20/KE).

### 4.2. Cell Culture

PBMCs were suspended in RPMI-1640 (supplemented with 1% penicillin/streptomycin (Sigma-Aldrich), 2 mM L-glutamate (Gibco, Waltham, MA, USA), and 10% heat-inactivated fetal bovine serum (FBS) (Gibco) to achieve a density of 1 × 10^6^ cells per mL, then in vitro cultures were established in 48-well sterile (non-pyrogenic) polystyrene flat-bottom plates (Corning, Tewksbury, MA, USA). The cells were cultured at 37 °C in a humidified atmosphere with 5% CO_2_, both in the presence and absence of β-glucans: zymosan A (Sigma-Aldrich, Cat. No. Z4250), prepared from *Saccharomyces cerevisiae* yeast cell wall (water-insoluble), and curdlan (Sigma-Aldrich, Cat. No. C7821), a water-insoluble linear β-(1,3)-glucan from *Alcaligenes faecalis*, with a purity of ≥98% (TLC). Both glucans were used at concentrations of 1 μg/mL or 10 μg/mL. Stock suspensions were prepared according to the manufacturer’s instructions. The cells were harvested after 72 h of incubation.

### 4.3. RNA Extraction, cDNA Synthesis, and Quantitative RT-PCR (RT-qPCR)

Total RNA was extracted from 5 × 10^6^ PBMCs using TRI Reagent^®^ (Sigma-Aldrich). The RNA concentration (A260) and purity (A260/A280) were measured with a NanoDrop™ 2000 spectrophotometer (Thermo Fisher Scientific, Waltham, MA, USA). The RNA samples were immediately frozen at −80 °C and stored until laboratory analysis. Following the manufacturer’s protocol, complementary DNA (cDNA) was synthesized using the High-Capacity cDNA Reverse Transcription Kit (Applied Biosystems, Foster City, CA, USA). The primers were designed using Primer3 Software and were confirmed through the Primer-BLAST online tool (https://www.ncbi.nlm.nih.gov/tools/primer-blast/ (accessed on 3 June 2025)) developed by NCBI and sourced from Genomed (Warsaw, Poland). The sequences of the primers are detailed in Table 2. The CFX Connect™ Real-Time PCR Detection System (Bio-Rad Laboratories, Hercules, CA, USA) was utilized to conduct RT-qPCR. Gene expression was quantified in duplicates using SsoAdvanced™ Universal SYBR^®^ Green Supermix (Bio-Rad Laboratories). The reaction mixture comprised 2 μL of forward and reverse primers (500 nM), 1 μL of cDNA template, 5 μL of SsoAdvanced™ Universal SYBR^®^ Green Supermix, and 2 μL of PCR-grade water (Gibco), resulting in a final volume of 10 μL. The PCR cycling conditions included an initial step at 95 °C for 30 s, followed by 40 cycles of denaturation at 95 °C for 10 s, and then annealing/extension at 60 °C for 10 s, in accordance with the manufacturer’s recommendations for the SsoAdvanced™ Universal SYBR^®^ Green Supermix (Cat. No. #1725271). The fold changes of the analyzed samples were calculated using the ΔΔCt method with CFX Maestro™ software (version 4.1.2433.1219) [61]. The reference gene, human *ACTB*, was employed to normalize the expression of mRNAs.

### 4.4. Treatment of PBMCs with Signaling Pathway Inhibitors

To analyze the role of the signaling molecules in curdlan- and zymosan-induced responses, purified PBMCs were prestimulated with a Syk inhibitor, R406 (InvivoGen, San Diego, CA, USA), at a concentration of 2 μM, a Dectin-1 antagonist, laminarin (Sigma-Aldrich), at a concentration of 100 μg/mL, a TLR4 antagonist, TAK-242 (Sigma-Aldrich), at a concentration of 5 ng/mL, and a p38 MAPK inhibitor, (SB203580) (InvivoGen), at a concentration of 10 μM, or with medium alone for 30 min in a humidified atmosphere containing 5% CO_2_ at 37 °C, before the primary procedure was conducted. The concentrations of the Syk inhibitor, laminarin, TAK-242, and the p38 MAPK inhibitor were initially based on the literature and were ultimately selected during preliminary experiments [62,63,64,65]. None of the chosen concentrations impacted PBMC viability, as determined by the trypan blue exclusion assay.

### 4.5. ROS Production Measurement

PBMCs suspended in RPMI-1640 medium were exposed to curdlan and zymosan at final concentrations of 1 or 10 μg/mL or to a medium alone for spontaneous ROS generation. The cells were then incubated for 72 h at 37 °C in a 5% CO_2_ humidified atmosphere. Next, Invitrogen™ CellROX™ Green Reagent (Thermo Fisher Scientific) was added at a final concentration of 5 μM, and the cells were incubated again at 37 °C for 30 min. Following this, a triple-wash procedure was performed using 1× PBS through centrifugation at 150× *g* for 5 min at 20 °C. Fluorescence intensity was measured using the FLUOstar Omega Microplate Reader (BMG Labtech, Ortenberg, Germany), with the excitation at 485 nm and emission at 520 nm. The ROS levels were quantified as the mean signal intensity (MSI).

### 4.6. Statistical Analysis

Statistical data analysis was conducted using STATISTICA 13.1 software (Statsoft Inc., Tulsa, OK, USA). Simple descriptive statistics (means and standard deviations (SD)) were computed for all continuous variables. The normality of distribution was assessed using the Shapiro–Wilk test. The Student’s *t*-test was used to identify statistically significant differences across all examined parameters, except for ROS production, for which the Mann–Whitney U test was employed. The significance level was established at *p* < 0.05 (two-sided).

## Figures and Tables

**Table 1:** Comparative characteristics of β-glucans used in the study.

	Zymosan A	Curdlan
Source Organism	*Saccharomyces cerevisiae*	*Alcaligenes faecalis*
Main Glycosidic Linkage	β-1,3- and β-1,6-glucan linkages	β-1,3-glucan only
Branching	Highly branched (due to β-1,6 side chains)	Linear (no branching)
Receptor Interaction	Influenced by Dectin-1	

**Table 2:** Sequences of primers used in the study.

Gene Name	Primer Sequence (5′-3′)
*ACTB*	Forward: CACCATTGGCAATGAGCGGTTC Reverse: AGGTCTTTGCGGATGTCCACGT
*CAT*	Forward: CCCTGAGGCATTTAGGCAGCTA Reverse: AGGTAGAGAGGTGGCTTAGGCT
*CCL2*	Forward: AGAATCACCAGCAGCAAGTGTCC Reverse: TCCTGAACCCACTTCTGCTTGG
*DECTIN1*	Forward: ACAATGCTGGCAACTGGGCTCT Reverse: AGAGCCATGGTACCTCAGTCTG
*DECTIN2*	Forward: TCAGTGAAGGGACAAAGGTGCC Reverse: CTCCCATCTCAACACAGTTCTGC
*GPX1*	Forward: GTGCTCGGCTTCCCGTGCAAC Reverse: CTCGAAGAGCATGAAGTTGGGC
*IL6*	Forward: AGACAGCCACTCACCTCTTCAG Reverse: TTCTGCCAGTGCCTCTTTGCTG
*IL17*	Forward: CGGACTGTGATGGTCAACCTGA Reverse: GCACTTTGCCTCCCAGATCACA
*IL22*	Forward: GTTCCAGCCTTATATGCAGGAGG Reverse: GCACATTCCTCTGGATATGCAGG
*IL23*	Forward: GAGCCTTCTCTGCTCCCTGATA Reverse: GACTGAGGCTTGGAATCTGCTG
*TGFB1*	Forward: TACCTGAACCCGTGTTGCTCTC Reverse: GTTGCTGAGGTATCGCCAGGAA
*TLR2*	Forward: CTTCACTCAGGAGCAGCAAGCA Reverse: ACACCAGTGCTGTCCTGTGACA
*TLR4*	Forward: CCCTGAGGCATTTAGGCAGCTA Reverse: AGGTAGAGAGGTGGCTTAGGCT
*TNF*	Forward: CTCTTCTGCCTGCTGCACTTTG Reverse: ATGGGCTACAGGCTTGTCACTC
*SOD1*	Forward: CTCACTCTCAGGAGACCATTGC Reverse: CCACAAGCCAAACGACTTCCAG

## Data Availability

The data presented in this study are available on request from the corresponding author. The data are not publicly available due to their inclusion in an ongoing research project.
